# Coping strategies of medical and non-medical personnel during the first wave of the pandemic of the novel coronavirus infection in Russia

**DOI:** 10.1192/j.eurpsy.2026.11016

**Published:** 2026-07-31

**Authors:** V. I. Rozhdestvenskiy, V. V. Titova, I. A. Gorkovaya, D. O. Ivanov, Y. S. Aleksandrovich

**Affiliations:** Department of Psychosomatics and Psychotherapy, Saint Petersburg State Pediatric Medical University, Saint Petersburg, Russian Federation

## Abstract

**Introduction:**

The pandemic of the novel coronavirus infection had a significant impact on all population groups in Russia. However, medical workers experienced the most significant stress, especially during the first wave of the pandemic, as they had to interact directly with patients, and some even had to work in so-called red zones. In such conditions, it was essential to investigate the coping strategies employed by medical workers to combat stress and to determine whether they differed from those used by non-medical workers. Coping strategies refer to cognitive, emotional, and behavioural strategies used to overcome stress and psychologically challenging situations.

**Objectives:**

The study aimed to identify the coping strategies used by medical workers and people not directly involved in medicine during the first wave of the COVID-19 pandemic in Russia.

**Methods:**

Data were collected between May and July 2020 using a Google form developed by the authors. The case comprised 97 Russian citizens who were not healthcare workers and 45 healthcare workers. The COPE-60 inventory was used to identify coping strategies. The instrument was adapted for use in the Russian context.

**Results:**

We found that healthcare workers, compared to non-medical professionals, more often used coping strategies such as ‘positive reinterpretation and personal growth’ (M = 13.62±2.85 — medical professionals, M = 12.08±3.14 — non-medical professionals, p < 0.05), ‘focusing on emotions and their active expression’ (M = 9.74±2.85 vs M = 7.90±2.83, p < 0.05), ‘use of instrumental social support’ (M = 11.20±3.06 vs M = 9.30±3.25, p < 0.05), ‘active coping’ (M = 12.93±3.18 vs M = 11.21±3.11, p < 0.05), ‘humour’ (M = 12.09±2.79 vs M = 10.91±3.34, p < 0.05), ‘use of emotional social support’ (M = 11.53±3.43 vs M = 9.62±3.34, p < 0.05), ‘use of “sedatives”’ (M = 6.82±3.14 vs M = 5.64±2.97, p < 0.05), ‘suppression of competing activities’ (M = 10.93±2.81 vs M = 8.96±3.07, p < 0.05) and ‘planning’ (M = 12.69±3.04 vs M = 11.20±3.15, p < 0.05).

**Conclusions:**

During the first wave of COVID-19 in Russia, medical workers employed productive coping strategies more frequently than non-medical workers, including seeking instrumental and emotional social support from others. At the same time, medical workers more often used alcohol, medicines or narcotics to combat stress, which could subsequently hurt their somatic health.

**Disclosure of Interest:**

None Declared

